# The down-regulation of the CYP2C19 gene is associated with aggressive tumor potential and the poorer recurrence-free survival of hepatocellular carcinoma

**DOI:** 10.18632/oncotarget.25178

**Published:** 2018-04-24

**Authors:** Ryo Ashida, Yukiyasu Okamura, Keiichi Ohshima, Yuko Kakuda, Katsuhiko Uesaka, Teiichi Sugiura, Takaaki Ito, Yusuke Yamamoto, Takashi Sugino, Kenichi Urakami, Masatoshi Kusuhara, Ken Yamaguchi

**Affiliations:** ^1^ Division of Hepato-Biliary-Pancreatic Surgery, Shizuoka Cancer Center, Shizuoka, Japan; ^2^ Medical Genetics Division, Shizuoka Cancer Center Research Institute, Shizuoka, Japan; ^3^ Division of Pathology, Shizuoka Cancer Center, Shizuoka, Japan; ^4^ Cancer Diagnostics Research Division, Shizuoka Cancer Center Research Institute, Shizuoka, Japan; ^5^ Regional Resources Division, Shizuoka Cancer Center Research Institute, Shizuoka, Japan; ^6^ Shizuoka Cancer Center Hospital and Research Institute, Shizuoka, Japan

**Keywords:** hepatocellular carcinoma, integrated gene expression profiling, CYP2C19 gene, down-regulation, recurrence-free survival

## Abstract

Project HOPE (High-tech Omics-based Patient Evaluation) began in 2014 using integrated gene expression profiling (GEP) of cancer tissues as well as diathesis of each patient who underwent an operation at our institution. The aim of this study was to clarify the association between the expression of cytochrome P450s (CYP) genes and recurrence of hepatocellular carcinoma (HCC). The present study included 92 patients. Genes with aberrant expression were selected based on a ≥10-fold difference in the expression between tumor and non-tumor tissues. The GEP analysis showed that the down-regulated genes in tumor tissue were CYP3A4 in 56 patients (61%), CYP2C8 in 44 patients (48%), CYP2C19 in 30 patients (33%), CYP2D6 in 11 patients (12%), CYP3A5 in 7 patients (8%) and CYP1B1 in 2 patients (2%). There was no patients with down-regulation of the CYP17A1 gene. A multivariate analysis revealed that the presence of microscopic portal invasion (hazard ratio [HR] 2.57, 95% confidence interval [CI] 1.30–5.05 *P* = 0.006), the presence of intrahepatic-metastasis (HR 3.09 95% CI 1.52–6.29 *P* = 0.002) and down-regulation of the CYP2C19 gene (HR 3.69 95% CI 1.83–7.46 *P* < 0.001) were independent predictors for the recurrence-free survival (RFS). The down-regulation of the CYP2C19 gene were correlated with the RFS in HCC.

## INTRODUCTION

Hepatocellular carcinoma (HCC) is the third-most common cause of cancer-related death worldwide, with exceedingly high rates in eastern/south-eastern Asia [1]. HCC usually develops in the setting of chronic inflammation due to viral hepatitis, excess alcohol consumption and metabolic diseases. The mechanism of liver carcinogenesis involves genetic, epigenetic, transcriptomic and metabolic changes that form its unique molecular fingerprint [2]. To reduce the cancer related death of HCC, elucidating the molecular mechanisms and developing novel biomarkers are essential, and many researchers have reported the results of whole-genome sequencing analyses [3–6] and microarray studies [7–11].

Project HOPE (High-tech Omics-based Patient Evaluation) began in 2014 using whole-exome sequencing and integrated gene expression profiling (GEP) of each cancer tissue as well as diathesis of each patient, who underwent surgery at Shizuoka Cancer Center Hospital [12]. As part of project HOPE, we previously reported the results of a GEP analysis for HCC, in which we extracted the top ten genes that were frequently up-regulated or down-regulated in tumor tissue from 820 cancer-related genes (SCC-820) [13]. Moreover, when the relationships between the expression of the extracted genes and overall survival and early recurrence were analyzed, cytochrome P450s (CYP) 3A4 was shown to be the only predictor of overall survival and early recurrence. We therefore concluded that CYP3A4 may be a novel biomarker for HCC [13]. Looking closely at the SCC-820 genes that were frequently up-regulated or down-regulated in tumor tissue, especially those that were down-regulated—in addition to CYP3A4—both CYP2C8 and CYP2C19 were included in the top thirty genes (Supplementary Table 1). We therefore focused on the CYP family in the present study. Although impaired activity and expression of CYP proteins and genes in HCC were reported [14], little is known about the prognostic impact of the CYP gene expression status for HCC [15–18]. The aim of this study was to clarify the association between the oncological behavior of HCC and expression of CYP genes and to identify a novel biomarker for the prognosis of HCC.

## RESULTS

### GEP analyses

**Figure 1:**
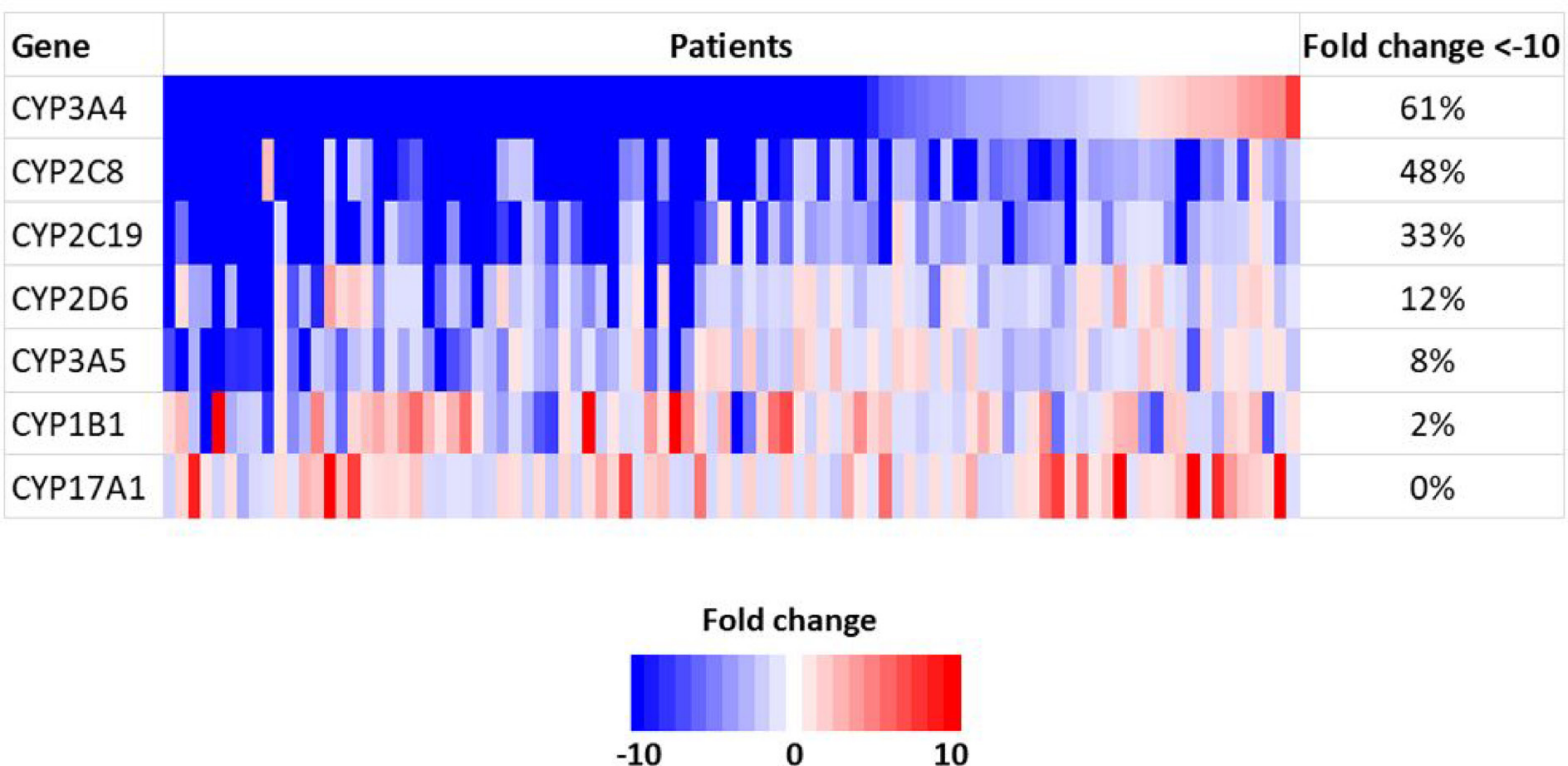
Gene expression of CYP family genes using a microarray analysis

Among the CYP family genes, seven genes, (CYP3A4, CYP2C8, CYP2C19, CYP2D6, CYP3A5, CYP1B1 and CYP17A1) were included in the SCC-820 genes [19]. The genes found to be down-regulated in tumor tissue were CYP3A4 in 56 patients (61%), CYP2C8 in 44 patients (48%), CYP2C19 in 30 patients (33%), CYP2D6 in 11 patients (12%), CYP3A5 in 7 patients (8%) and CYP1B1 in 2 patients (2%). There was no patients with down-regulation of the CYP17A1 gene (Figure 1). We used these six down-regulated genes as candidate novel biomarkers for predicting the recurrence-free survival (RFS) in patients with HCC.

### Prognostic factors for the RFS

**Figure 2:**
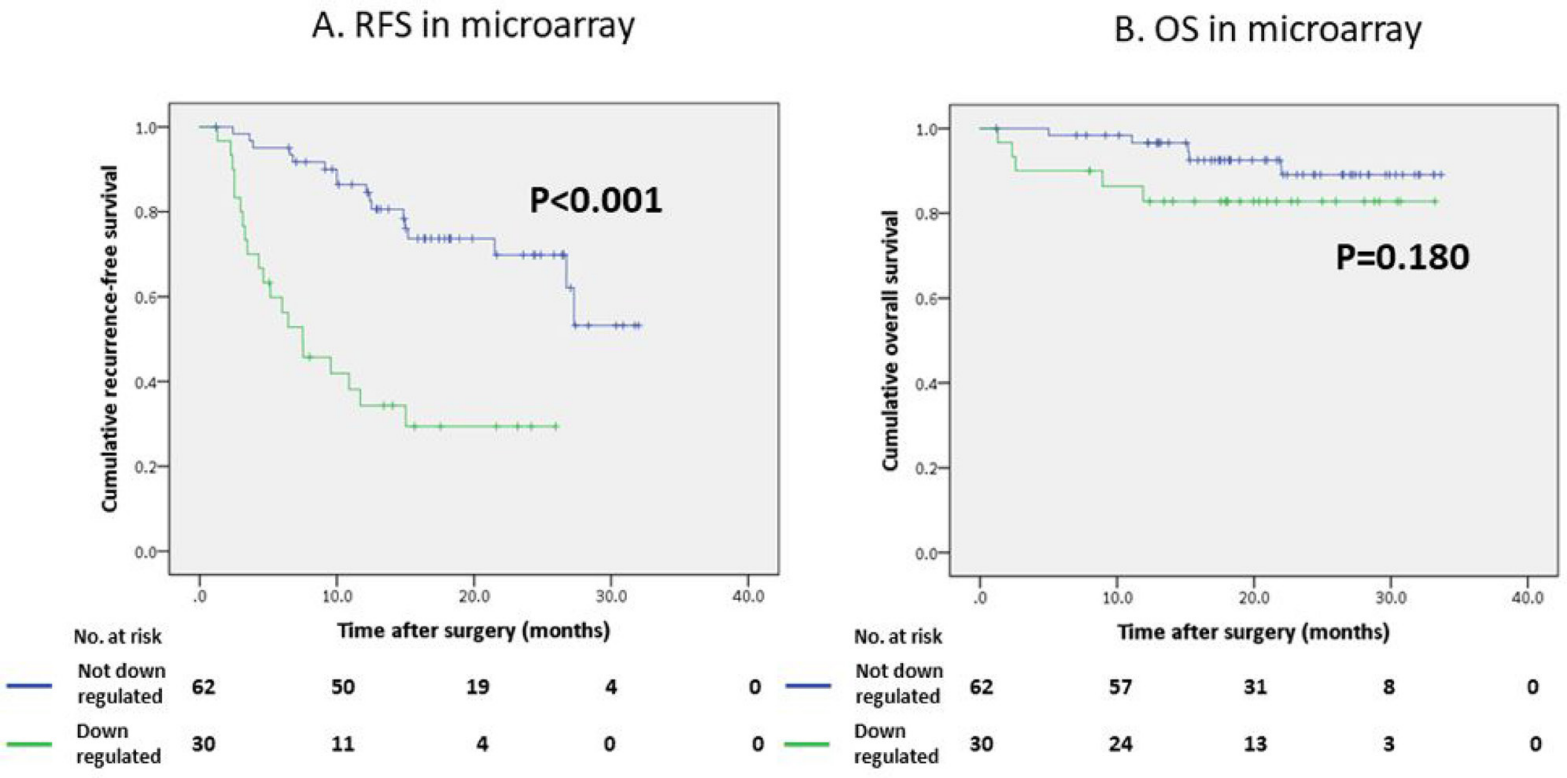
Survival curves of patients who underwent hepatectomy using the Kaplan-Meier method (**A**) Recurrence-free survival (RFS) curve according to the CYP2C19 gene expression in a microarray analysis. (**B**) Overall survival (OS) curve according to the CYP2C19 gene expression in a microarray analysis.

In the univariate analysis, des-gamma-carboxy prothrombin (DCP) < 40 mAU/mL (*P* = 0.010), a maximum tumor diameter ≥ 50 mm (*P* = 0.012), multiple tumors (*P* = 0.018), the presence of microscopic portal invasion (*P* < 0.001), the presence of microscopic venous invasion (*P* = 0.007), the presence of microsatellite lesions (*P* < 0.001), Union for International Cancer Control (UICC) stage ≥ II (*P* < 0.001), down-regulation of the CYP2C8 gene (*P* = 0.003), down-regulation of the CYP2C19 gene (*P* < 0.001), down-regulation of the CYP2D6 gene (*P* < 0.001), down-regulation of the CYP3A5 gene (*P* = 0.017), and down-regulation of the CYP1B1 gene (*P* = 0.004) were significant predictors of the RFS. The multivariate analysis to identify novel biomarkers revealed that the presence of microscopic portal invasion (hazard ratio [HR] 2.57, 95% confidence interval [CI] 1.30–5.05 *P* = 0.006), the presence of microsatellite lesions (HR 3.09 95% CI 1.52–6.29 *P* = 0.002) and down-regulation of the CYP2C19 gene (HR 3.69 95% CI 1.83–7.46 *P* < 0.001) were independent predictors of the poorer RFS (Table 1). The RFS was significantly worse in the patients with down-regulation of the CYP2C19 gene than in those without down-regulation of the CYP2C19 gene (Figure 2A, *P* < 0.001).

**Table 1:** Prognostic factors for recurrence-free survival by univariate and multivariate analysis

Variables			Univariate	Multivariate	
	Number	2-years survival (%)	*P*	Hazard ratio (95% Confidence interval)	*P*
AFP			0.254		
<10 ng/ml	44	51.4			
≥10 ng/mL	48	63.2			
DCP			0.010		0.180
<40 mAU/mL	15	38.9		1.89 (0.75–4.78)	
≥40 mAU/mL	77	60.8		1	
Histologic differentiation			0.104		
Well	17	72.1			
Others	75	53.4			
Size			0.012		0.204
<50 mm	48	66.4		1	
≥50 mm	44	46.0		1.65 (0.76–3.54)	
Tumor number			0.018		0.697
solitary	72	61.3		1	
multiple	20	39.7		1.43 (0.60–3.46)	
Microscopic portal invasion			<0.001		0.006
absent	69	67.5		1	
present	23	25.3		2.57 (1.30–5.05)	
Microscopic venous invasion			0.007		0.885
absent	70	64.5		1	
present	22	33.9		1.08 (0.40–2.88)	
Microsatellite lesions			<0.001		0.002
absent	74	65.4		1	
present	18	22.1		3.09 (1.52–6.29)	
Tumor stage			<0.001		0.286
I	46	77.2		1	
II + III	46	34.4		2.11 (0.53–8.33)	
CYP3A4 gene			0.221		
Down-regulated	56	58.1			
Not down-regulated	36	55.0			
CYP2C8 gene			0.003		0.947
Down-regulated	44	43.3		1.03 (0.41–2.60)	
Not down-regulated	48	68.4		1	
CYP2C19 gene			<0.001		<0.001
Down-regulated	30	29.4		3.69 (1.83–7.46)	
Not down-regulated	62	69.8		1	
CYP2D6 gene			<0.001		0.719
Down-regulated	11	12.1		1.28 (0.33–4.98)	
Not down-regulated	81	62.7		1	
CYP3A5 gene			0.017		0.298
Down-regulated	7	21.4		1.79 (0.60–5.36)	
Not down-regulated	85	59.6		1	
CYP1B1 gene			0.004		0.054
Down-regulated	2	Not available		4.52 (0.98–20.83)	
Not down-regulated	90	58.0		1	

AFP, alpha-fetoprotein; DCP, des-gamma-carboxy prothrombin; CYP3A4, cytochrome P450, family 3, subfamily A, polypeptide 4; CYP2C8, cytochrome P450, family 2, subfamily C, polypeptide 8; CYP2C19, cytochrome P450, family 2, subfamily C, polypeptide 19; CYP2D6, cytochrome P450, family 2, subfamily D, polypeptide 6; CYP3A5, cytochrome P450, family 3, subfamily A, polypeptide 5; CYP1B1, cytochrome P450, family 1, subfamily B, polypeptide 1.

**Figure 3:**
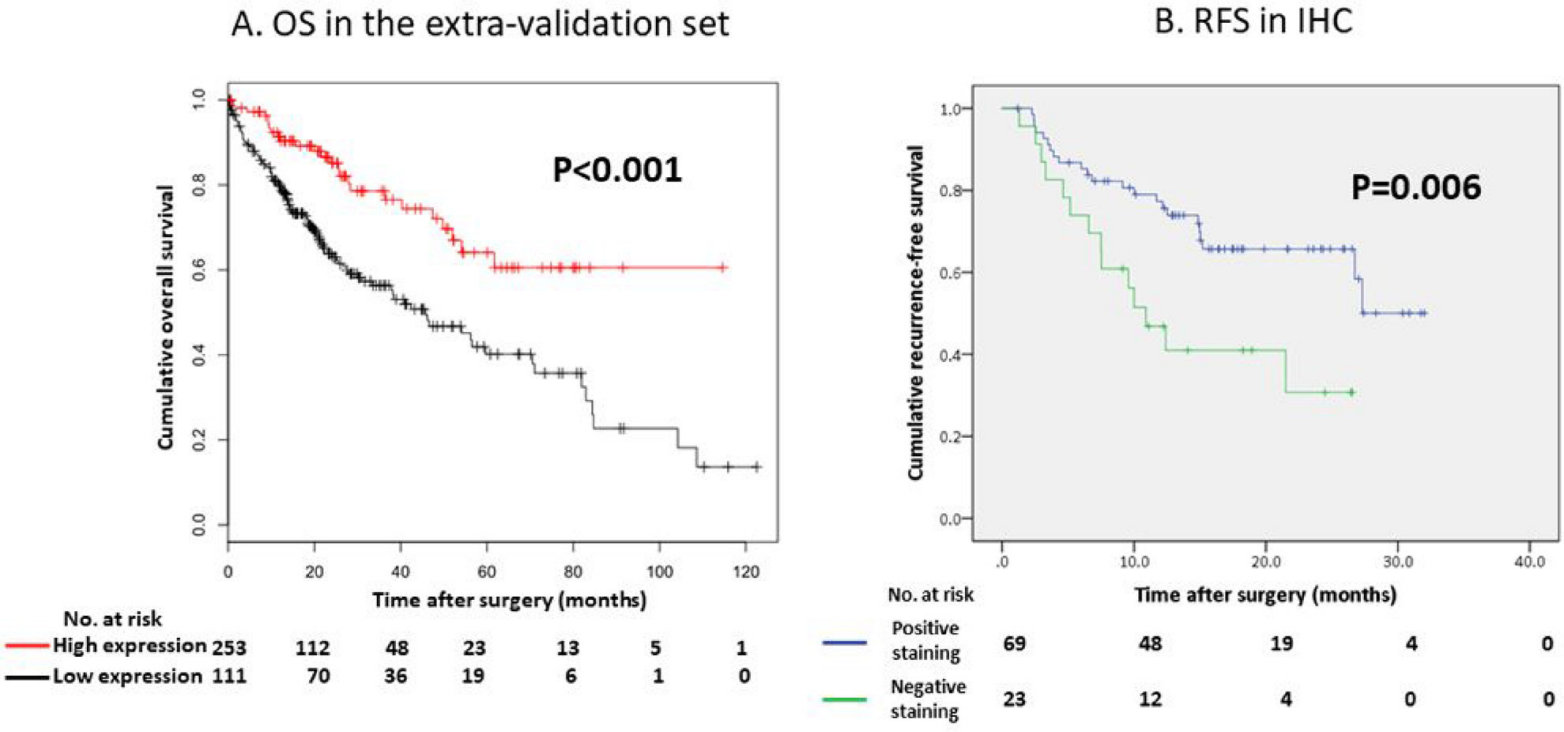
**(A**) Overall survival (OS) curve according to the CYP2C19 gene expression in the extra-validation set. (**B**) Recurrence-free survival (RFS) curve according to the IHC status of CYP2C19 protein in our study.

In contrast, the regulation status of the CYP2C19 gene did not affect the overall survival (OS) of our study population (Figure 2B, *P* = 0.180); however the OS of patients with low CYP2C19 gene expression levels was significantly worse than that of the patients with high CYP2C19 gene expression levels in the extra validation set from the Kaplan-Meier Plotter (KM Plotter) database (Figure 3A, *P* < 0.001).

### A comparison of the clinicopathological factors according to the CYP2C19 gene expression

The frequency of well-differentiated HCC in the patients with down-regulation of the CYP2C19 gene was significantly lower than in those without down-regulation of the CYP2C19 gene (*P* = 0.009). The tumor size in the patients with down-regulation of the CYP2C19 gene was significantly greater than in those without down-regulation of the CYP2C19 gene (*P* = 0.005). The frequency of the microscopic portal invasion (*P* = 0.005), microscopic venous invasion (*P* = 0.012) and UICC stage ≥ II (*P* < 0.001) in the patients with down-regulation of the CYP2C19 gene were significantly higher than in those without down-regulation of the CYP2C19 gene (Table 2).

**Table 2:** Demographics of patients according to CYP2C19 gene expression

	Down regulated (*n* = 30)	Not down regulated (*n* = 62)	*P*
Etiology (viral)	16 (53%)	30 (48%)	0.656
HBsAg (positive)	7 (23%)	8 (13%)	0.204
Anti-HCV Ab (positive)	9 (30%)	22 (36%)	0.602
Cirrhosis (present)	5 (17%)	12 (19%)	0.755
Differentiation (well)	1 (3% )	16 (26%)	0.009
Tumor number (multiple)	10 (33%)	10 (19%)	0.061
Size (mm)^#^	75 (10–180)	35 (9–160)	0.005
Growth pattern (infiltrative)	1 (3%)	4 (6%)	1.000
Microscopic portal invasion (present)	13 (43%)	10 (16%)	0.005
Microscopic venous invasion (present)	12 (40%)	10 (16%)	0.012
Microsatellite lesions (present)	8 (27%)	10 (16%)	0.232
Tumor stage (II + III)	23 (77%)	23 (37%)	<0.001

HBsAg, hepatitis B surface antigen; HCV, hepatitis C virus; Ab, antibody;

CYP2C19, cytochrome P450, family 2, subfamily C, polypeptide 19.

^#^The value indicates the median (range).

### Relationship between CYP2C19 gene expression status and recurrence status

During the study period, recurrence was observed in 20 patients with the down-regulation of the CYP2C19 gene and 17 patients without the down-regulation of the CYP2C19 gene. There were no significant differences between the two groups with regard to the site of recurrence or the number of recurrent sites. The median time to recurrence in the patients with the down-regulation of the CYP2C19 gene was significantly shorter than that in patients without the down-regulation of the CYP2C19 gene (4.9 months vs. 12.2 months, respectively; *P* = 0.002).

### Association between the expression of the CYP2C19 gene in a microarray analysis and reverse transcription polymerase chain reaction (RT-PCR) of tumor tissue

**Figure 4:**
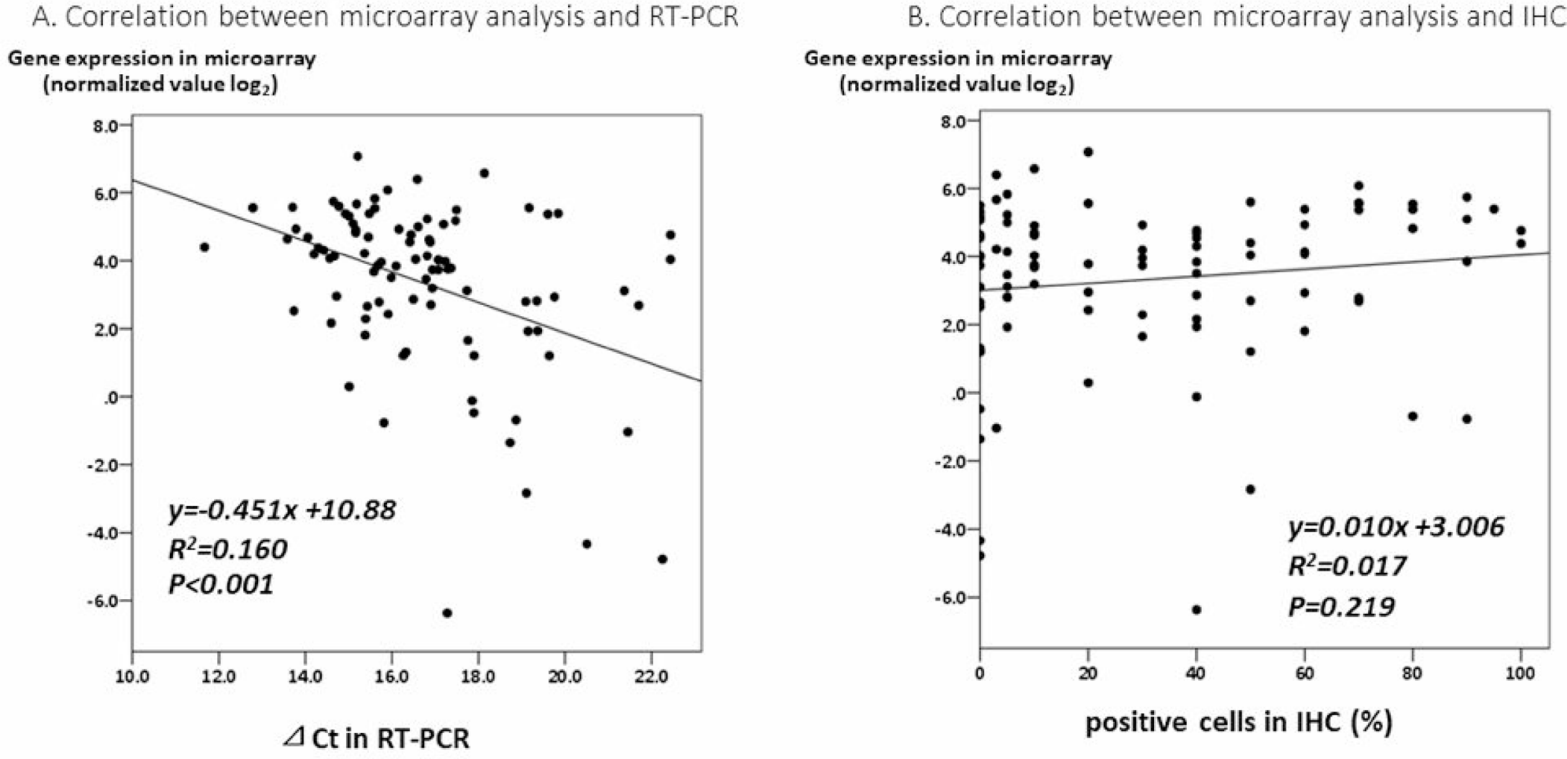
**(A**) A correlation analysis between the expression of the CYP2C19 gene in a microarray analysis and by RT-PCR of tumor tissue. (**B**) A correlation analysis between the expression of CYP2C19 gene in a microarray analysis and the IHC staining of CYP2C19 protein of tumor tissue.

To verify the results of the GEP analysis, we evaluated the association between the expression of the CYP2C19 gene in the microarray analysis and those in RT-PCR of the tumor tissue using Spearman’s correlation coefficient. Although the correlation coefficient was relatively low (*R*^2^ = 0.16), positive correlations were found (Figure 4A, *P* < 0.001)

### Association between immunohistochemical staining (IHC) of CYP2C19 protein and RFS

**Figure 5:**
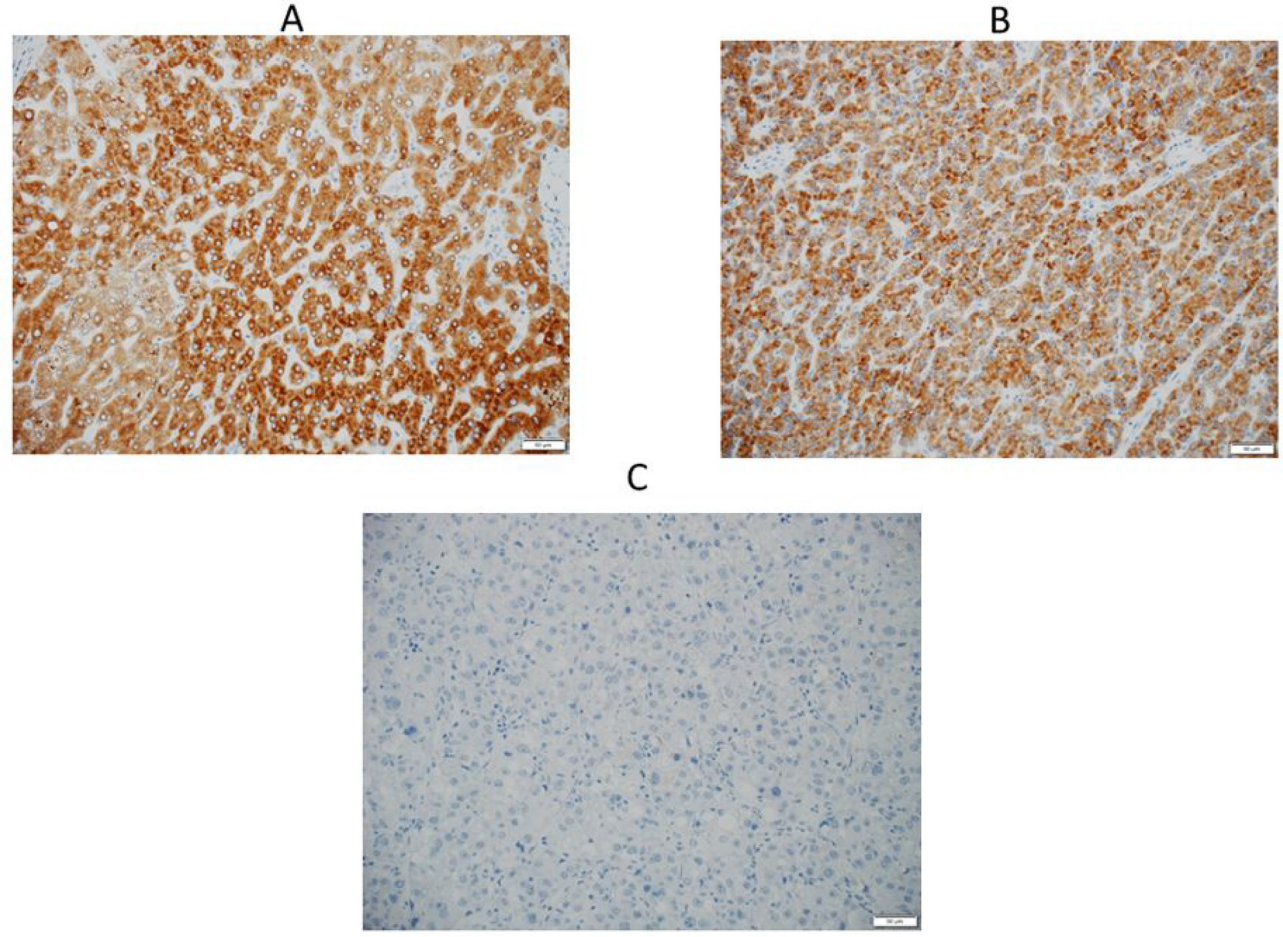
**(A**) An IHC analysis of CYP2C19 in non-tumor tissue, and cytoplasm of the hepatocyte is positively stained. (**B**) An IHC analysis of CYP2C19 in tumor tissue, which showed positive staining in the cytoplasm. (**C**) An IHC analysis of CYP2C19 in tumor tissue, which showed negative staining.

In the IHC analysis, CYP2C19 protein was stained in the cytoplasm of non-tumor cells (Figure 5A). Figure 5B and 5C show positive and negative staining in the cytoplasm of tumor cells, respectively. The optimum cut-off value of staining for CYP2C19 was 5% when using the minimum *P* value approach. The staining for CYP2C19 protein was classified according to the percentage of positive cells: staining in ≥ 5% of tumor cells was regarded as positive and in < 5% of cells as negative. Positive staining were observed in tumor cells of 69 patients (75%) and negative staining were observed in 23 patients (25%). Although the IHC analysis did not reveal a significant correlation between positive staining of CYP2C19 protein in tumor tissue and the expression of CYP2C19 gene in the microarray analysis (Figure 4B, *P* = 0.219), the cumulative RFS rate in patients with negative staining of CYP2C19 protein was significantly poorer than in patients with positive staining of CYP2C19 (Figure 3B, *P* = 0.006).

## DISCUSSION

In the present study, we performed an integrated GEP analysis of CYP genes included in SCC-820 for patients with HCC. We analyzed the relationship between the expression of CYP genes and the prognosis and found that the down-regulation of the CYP2C19 gene was an independent predictor of the RFS. Although there were no significant differences in the multivariate analysis, down-regulation of the CYP2C8, CYP2D6, CYP3A5 and CYP1B1 genes was a significant predictor of the RFS in the univariate analysis. Correlations of mRNA expression between several major CYP genes for tumor tissue of HCC have been reported, which may explain our results [14]. To verify the results of the microarray analysis, we performed RT-PCR and IHC. RT-PCR supported the findings of the microarray analysis. In the IHC analysis, the cumulative RFS rate in patients with negative staining of CYP2C19 protein was significantly poorer than in those with positive staining, which was the same result as obtained in the microarray analysis. Therefore, we identified CYP2C19 as a novel, potential clinically useful biomarker for the RFS of HCC. There are no reports of integrated microarray analyses of the GEP for patients with HCC focusing on the expression of CYP genes and their prognostic impact. The present study is the first report to describe the frequency of down-regulation of CYP genes in microarray analyses and its prognostic impact for the RFS.

The present study showed that tumors are more aggressive with regard to differentiation, size, microscopic portal invasion, microscopic venous invasion and stage in patients with down-regulation of the CYP2C19 gene than in those without down-regulation. Several papers have reported that tumor differentiation [20], tumor size [21], microscopic portal invasion [22], microscopic venous invasion [22] and tumor stage [23] are associated with a poor prognosis, and these findings might be related to the poor RFS of patients with down-regulation of the CYP2C19 gene compared to those without such down-regulation. In addition, the fact that the time to recurrence in the patients with down-regulation of the CYP2C19 gene being significantly shorter than in those without down-regulation might be related to the poorer RFS.

CYPs are responsible for about 75% of drug metabolism and for the metabolism of a huge amount of dietary constituents and endogenous chemicals [24]. Humans have 59 active CYP genes, and CYP2C19 is mainly expressed in the liver; its enzymes are involved in the metabolism of about 20% of currently used drugs [25]. Genetic variants of CYP2C19 DNA are polymorphic and the common CYP2C19*2 and CYP2C19*3 alleles have nucleotide mutations that cause a splicing error and generation of a termination codon, which result in enzyme deficiency in non-tumor tissue of the liver [26]. Individuals with such mutations are called poor metabolizers (PMs), and this status is most common among Asians (35% allele frequency) [27]. Chau *et al.* reported that PM status of CYP2C19 is associated with an increased incidence of HCC development and suggested that CYP2C19 plays a role in the detoxification of carcinogens [28]. Wang *et al.* reported a comprehensive meta-analysis that examined the CYP2C19 polymorphisms and the relationship with several cancers, including HCC, and found that PM status of CYP2C19 most likely contributes to cancer susceptibility. They also suggested that CYP2C19 influences the metabolism, particularly detoxification of the carcinogens as a tumor suppressor [29]. The down-regulation of CYP2C19 might therefore be associated with a lower metabolism of carcinogens, which leads to higher exposure of carcinogens. As a result, carcinogenesis and proliferation easily occurs in patients with down-regulation of the CYP2C19 gene, thereby leading to an aggressive manifestation and a poor prognosis of HCC.

Although the correlation coefficient was relatively low (*R*^2^ = 0.16), significant correlations between the expression of the CYP2C19 gene in microarray analyses and the expression of the CYP2C19 gene in RT-PCR were found. These suggest that the results of GEP using the microarray were correct. However, microarray analyses and RT-PCR are difficult to perform in daily clinical practice. The present study therefore confirmed the expression of the CYP2C19 protein using IHC, and the cumulative RFS rate in patients with negative staining of CYP2C19 protein was significantly poorer than that in patients with positive CYP2C19 staining, which was similar to the results of the microarray analysis. No significant correlations were found between the CYP2C19 protein staining status in the IHC analysis and the expression of the CYP2C19 gene in the microarray analysis, which might be a weak point of the present study. Zhang *et al.* reported that the expression of CYP2C subfamily proteins, such as CYP2C19, CYP2C8 and CYP2C9, was partially post-transcriptionally regulated by microRNA-103 and 107 [30]. They reported that there were no correlation between the protein levels of CYP2Cs and the mRNA levels of CYP2Cs in human liver samples. This may be why the expression of the CYP2C19 gene in the microarray analysis and the IHC staining status of the CYP2C19 protein were not significantly correlated. Although these mechanisms should be verified in the future, we have confirmed the utility of CYP2C19 protein as a novel biomarker for predicting the prognosis in daily clinical practice. At the time of writing an appropriate adjuvant chemotherapy regimen for HCC remains to be established, and a shortened follow-up interval may be beneficial for patients with negative CYP2C19 protein staining as these patients were potentially high risk of recurrence. Moreover, to improve the prognosis of HCC, the proper identification of HCC patients who may benefit from adjuvant chemotherapy will be necessary.

Regarding the mechanisms underlying the down-regulation of the CYP2C19 gene in the tumor tissue of HCC, the expression of the CYP2C19 gene was regulated by several nuclear receptors and transcription factors, including the constitutive androstane receptor (CAR), pregnane X receptor, glucocorticoid receptor, and hepatocyte nuclear factor-3g. Of these, CAR seemed to play a central role and had a strong association with the basal expression of CYP2C19 [31]. Promoter hyper-methylation has been reported to result in repression of CAR expression, which induced the down-regulation of the CYP2C19 gene, in human non-cancerous liver and human hepatoma cell lines [32]. In contrast, however, Burns *et al.* reported that the relationships between already known transcription factors for CYP2C19 and the expression of CYP2C19 gene in human liver samples accounted for less than 70% of the variability in CYP2C19 mRNA levels. They suggested that an as-yet-un-identified master regulator of CYP2C19 transcription may itself be a target of epigenetic control [33]. Although the precise mechanisms underlying the down-regulation of the CYP2C19 gene were not elucidated in human HCC tissue, several mechanisms, especially epigenetic modification, such as promoter hyper-methylation of CAR might repress the expression of the CYP2C19.

There are several limitations associated with the present study. First, among the numerous active CYP genes, we focused on only the seven major genes included in 820 cancer-related genes. Second, the follow-up period was relatively short and the number of the patients was relatively small in the present study. For these reasons, there was no significant association between OS and the CYP2C19 expression status and to compensate for this limitation, a further analysis was performed using the extra-validation set. Further prospective multi-institutional studies are therefore needed to validate the results of the present study objectively.

In conclusion, the down-regulation of the CYP2C19 gene and protein were found to be correlated with the RFS in HCC.

## MATERIALS AND METHODS

### Subjects

Surgically resected tumor specimens were obtained from 92 consecutive patients who underwent curative resection for HCC at the Division of Hepato-Biliary-Pancreatic Surgery of Shizuoka Cancer Center Hospital between January 2014 and October 2016 and had enrolled in Project HOPE. All pathological slides of specimens from those patients were reviewed. Clinical and pathological data were collected from our prospectively recorded database. The patient characteristics have been previously described [13]. Hepatitis B virus (HBV) and hepatitis C virus (HCV) infection were defined based on the detection of HBsAg and HCVAb, respectively. The tumor stage was also assessed based on the seventh edition of the UICC classification [34]. Ethical approval for all experimental protocols and study was obtained from the institutional review board at the Shizuoka Cancer Center (Authorization Number: 25–33). Written informed consent was obtained from all patients enrolled in the study. All experiments using clinical samples were carried out in accordance with the approved guidelines.

### Clinical samples

Tumor tissue samples with sizes corresponding to weights of ≥0.1 g were dissected from resected specimens, along with samples of surrounding normal tissue. The areas from which tumor samples were dissected were visually assessed as containing ≥50% tumor content. For the RNA analysis, tissue samples were submerged in RNAlater solution (Thermo Fisher Scientific), minced, and stored at 4° C before RNA extraction. In cases involving multiple tumors, tissue samples were collected from the largest tumor.

### RNA isolation

Total RNA was extracted from approximately 10 mg of minced tissue samples using the miRNeasy Mini Kit (Qiagen) as described previously [19]. RNA samples with RNA integrity number ≥6.0 were used for the microarray analysis.

### The GEP analysis

The microarray analysis was performed as described previously [19]. In brief, total RNA (100 ng) was fluorescence-labeled and hybridized to the SurePrint G3 Human Gene Expression 8 × 60 K v2 Microarray (Agilent Technologies). The microarray analysis was performed in accordance with the MIAME guidelines [35]. Data analysis was performed using the GeneSpring GX software program (Agilent Technologies) and Microsoft Excel. Raw signal intensity values were log-transformed and normalized to the 75th percentile. The fold-change between the tumor and non-tumor tissues from the same patient was calculated using the normalized intensity values. Genes with expression levels with > 10-fold in tumor tissues were defined as up-regulated and genes with expression levels with < -10-fold in tumor tissues were defined as down-regulated [19]. For external data validation, the KM Plotter database, an integrated dataset including 364 patients from three major cancer research centers (Berlin, Bethesda and Melbourne datasets), was accessed at http://kmplot.com/analysis/[36]. This dataset was used to verify the prognostic impact of the CYP2C19 gene expression status on OS.

### RT-PCR for the mRNA analysis

Quantitative mRNA levels were determined using real-time RT-PCR with the Applied Biosystems 7900 HT Sequence Detection System (Applied Biosystems), a TaqMan Gene Expression assay for human CYP2C19 (assay ID Hs00426380; Applied Biosystems), and a Eukaryotic 18S rRNA Endogenous Control (Applied Biosystems) as an endogenous control. cDNA was generated using 100 ng of the total RNA and a High-capacity cDNA Reverse Transcription Kit (Applied Biosystems). RT-PCR was carried out in a total volume of 20 μL using 100 ng of cDNA, TaqMan Fast Advanced Master Mix (Applied Biosystems), and the respective TaqMan reagents for target genes. The conditions for amplification were 95° C for 20 s followed by 40 cycles at 95° C for 1 s and 60° C for 20 s. Samples were analyzed in triplicate as biological replicates. The levels of CYP2C19 mRNA were defined from the cycle threshold (Ct). The Ct was normalized with reference to the level of 18S rRNA in each sample using the comparative Ct method, and ⊿Ct was defined as the difference in the Ct values for CYP2C19 mRNA and 18S rRNA [37].

### The IHC analysis

All of the resected specimens were fixed in 10% formalin, dehydrated and embedded in paraffin. Paraffin sections of 3-µm thickness containing representative well-preserved HCC samples were used for the IHC analysis. IHC was performed using the Bond III automated stainer and BOND Polymer Refine Detection kit (Leica Biosystems). The sections were pretreated with epitope retrieval BOND1 for 20 min at 100° C and then reacted with anti-CYP450 2C19 rabbit polyclonal antibody at 1.200 dilution (HPA015066, Sigma-Aldrich). After reaction with diaminobenzidine chromogen, the sections were counterstained with hematoxylin.

To avoid bias, two independent pathologists evaluated the specimens in a blinded manner as follows. The intensity of the CYP2C19 protein expression in tumor cells was described as the percentage of stained cells. The optimum cut-off CYP2C19 protein staining value for dividing the patients into 2 groups was determined using the minimum *P* value approach. The optimum cut-off point for dividing the patients based on their recurrence-free survival was also determined using the minimum *P* value approach, which was performed using a log-rank test [38, 39].

### Statistical analyses

The categorical variables were compared using the chi-squared test or Fisher’s exact test, as appropriate. Continuous variables were compared using the Mann-Whitney *U* test. The cumulative RFS were analyzed using the Kaplan–Meier method and compared using the log-rank test. A Cox proportional hazards model was used for the univariate and multivariate analyses, and all factors found to be significant predictors of the RFS (*P* < 0.10) in the univariate analysis were entered into the multivariate analysis. When converting continuous variables to categorical variables, the AFP and DCP levels were defined as the upper limit of normal at our institution. The cutoff value for the tumor size was determined based on the seventh edition of the UICC classification [34]. The multivariate analysis was performed via the logistic regression method using a backward stepwise selection model. All statistical analyses were performed using the SPSS 24.0 software package (SPSS, Inc., Chicago, IL, USA), and *P* values of < 0.05 in 2-tailed tests were considered to be significant.

## SUPPLEMENTARY MATERIALS TABLE





## Abbreviations

HCC: hepatocellular carcinoma
Project HOPE: high-tech omics-based patient evaluation
GEP: gene expression profiling
AFP: alpha-fetoprotein
DCP: des-gamma-carboxy prothrombin
UICC: Union for International Cancer Control
RT-PCR: reverse transcription polymerase chain reaction
Ct: cycle threshold
IHC: immunohistochemical
RFS: recurrence-free survival
PMs: poor metabolizers
CAR: constitutive androstane receptor
