# Supplementary material for: The down-regulation of the CYP2C19 gene is associated with aggressive tumor potential and the poorer recurrence-free survival of hepatocellular carcinoma
Source: Oncotarget. 2018 Apr 24;9(31):22058–68. doi: 10.18632/oncotarget.25178 (PMC5955155; doi:10.18632/oncotarget.25178)
Supplement: Supplementary file 1 [file oncotarget-09-22058-s001.pdf]

The down-regulation of the CYP2C19 gene is associated with aggressive tumor potential and the poorer recurrence-free survival of hepatocellular carcinoma

SUPPLEMENTARY MATERIALS

Supplementary Table 1: Each fold change values of top 30 up-regulated genes and top 30 down-regulated genes in microarray analysis. See Supplementary_ Table_1
